# Rosiglitazone attenuates hypoxia-induced renal cell apoptosis by inhibiting NF-κB signaling pathway in a PPARγ-dependent manner

**DOI:** 10.1080/0886022X.2022.2148539

**Published:** 2022-11-24

**Authors:** Jun-Yu Wei, Miao-Yue Hu, Xiu-Qi Chen, Feng-Ying Lei, Jin-Shuang Wei, Jie Chen, Xuan-Kai Qin, Yuan-Han Qin

**Affiliations:** a Department of Pediatrics, Guangxi Medical University, Nanning, China; b Department of Pediatrics, The First Affiliated Hospital, Guangxi Medical University, Nanning, China

**Keywords:** Rosiglitazone, PPARγ, Hypoxia renal injury, NF-κB, apoptosis

## Abstract

**Background:**

In recent years, peroxisome proliferator-activated receptor γ (PPARγ) has been found to be closely associated with hypoxia renal disease. The aim of this study was to investigate the relationship between rosiglitazone and mitochondrial apoptosis in renal tissue and its associated mechanisms.

**Methods:**

Twenty-four male Sprague-Dawley rats were randomly divided into three groups (*n* = 8 in each): normal control group, hypoxia injury group (equal volume of 0.9% saline), and PPARγ agonist group (Rosiglitazone, 10 mg/kg · d, intraperitoneally). The hypoxia injury group and PPARγ agonist group were placed in a hypoxia chamber and the simulated altitude was set at 7,000 m for 7 days. Blood and kidney samples were collected after 7 days. The quantitative real-time polymerase chain reaction and Western blot methods were used to determine the expression of PPARγ, nuclear factor kappa-B (NF-κB), B-cell lymphoma-2 (Bcl-2), and Bax.

**Results:**

The results showed that compared with the normal control group, the renal tissue of rats after hypoxia was severely damaged, as shown by massive renal tubular epithelial cell degeneration and detachment, and renal tubular dilation. The NF-κB protein expression significantly increased, the Bcl-2 protein and mRNA expression significantly decreased, and Bax protein and mRNA expression significantly increased (*p* < .05 for all). Renal injury was much less severe in the PPARγ agonist group compared to the hypoxia injury group.

**Conclusions:**

Rosiglitazone can alleviate hypoxia renal injury, with the possible mechanism involving attenuation of apoptosis by inhibiting the activation of the NF-κB signaling pathway in a PPARγ-dependent manner and increasing Bcl-2 and decreasing Bax expression.

## 1. Introduction

The kidney is highly susceptible to hypoxia-induced injury, and renal tissue hypoxia is considered one of the important factors in the pathophysiology of acute kidney injury and chronic kidney disease [1,2], which can lead to a vicious cycle such as tubular injury, vascular rarefaction, and fibrosis, thereby exacerbating hypoxia [3,4]. Renal hypoxia has been found to lead to renal tubular epithelial cells (RTEC) apoptosis [5]. Mitochondrial apoptosis is one of the classical pathways of apoptosis, and activation of the mitochondrial apoptotic pathway can start apoptosis [6]. B-cell lymphoma-2 (Bcl-2) family plays an important role in the mitochondrial apoptosis pathway [7]; especially the Bax family can promote cytochrome-c release from mitochondrial intermembrane space, thereby promoting the activation of caspase family members and ultimately leading to apoptosis [8–10]. Apoptosis has been found to be involved in renal injury in related renal diseases, such as renal ischemia-reperfusion [11], chronic kidney disease [12], and diabetic nephropathy [13]. However, there are few reports on the effects of systemic hypobaric hypoxia on renal structure and function.

Peroxisome proliferator-activated receptors (PPARs) are nuclear transcription factors that can be divided into three subtypes (*α*, *β* and *γ*) according to different coding genes; the distribution and function of each subtype are different *in vivo* [14]. PPARγ is expressed not only in adipose tissue, vascular smooth muscle, and other tissues but also selectively in renal medullary collecting ducts, glomeruli, and pelvic urinary tract epithelium [15,16]. PPARγ has been reported to act mainly in the glomerulus [17], and rosiglitazone improves glomerular hyperfiltration in patients with incipient diabetic nephropathy [18]. In addition, PPARγ is involved in kidney disease by participating in cell proliferation, apoptosis, inflammation, oxidative stress, as well as lipid metabolism, and activation of PPARγ improves kidney injury [19]. Our previous study showed that the agonist rosiglitazone could up-regulate the expression of PPARγ in the hypoxia-induced RTEC model, thus alleviating RTEC injury [20], which suggests that PPARγ plays an important role in hypoxia renal injury. Alternatively, it has also been reported that PPARγ may exert renal protection effects by inhibiting apoptosis in acute kidney injury [21,22]. However, the role and mechanism of PPARγ in the mitochondrial apoptosis pathway in renal tissue of rats with renal injury induced by hypobaric hypoxia are still rarely reported. Therefore, our aim was to investigate the relationship between PPARγ and mitochondrial apoptosis in kidney tissue and its mechanism.

## 2. Materials and methods

### 2.1. Animals

Specific pathogen-free (SPF) Sprague-Dawley (SD) male rats weighing around 120–150 g were purchased from the Laboratory Animal Center of Guangxi Medical University and housed in SPF-grade laboratories. The experiment started after one week of acclimatization. All animal experiments were approved by The Animal Care & Welfare Committee of Guangxi Medical University (Project Proposal number 202107003). These experiments also follow ARRIVE guidelines and are following the U.K. Animals (Scientific Procedures) Act,1986 and related guidelines, EU Directive 2010/63/EU for animal experiments, and the National Research Council’s Guide for the Care and Use of Laboratory Animals.

### 2.2. Animal grouping and model preparation

After one week of adaptive feeding, 24 male SD rats were divided randomly into three groups of eight rats each: normal control group, hypoxia injury group and PPARγ agonist group. The model group rats were placed in a hypobaric hypoxia chamber (Shanghai Tawang Intelligent Technology Co. Ltd., China) and the hypoxia environment was simulated by evacuating the air in the chamber using a powerful vacuum pump. Referring to the hypobaric hypoxia model conditions applied in the study of Ning Li et al. [23], the simulated altitude was 7,000 m and the duration of hypoxia was seven days. The rats were free to eat and drink. The chamber was opened for 0.5 h every day for drug administration and bedding material change. The PPARγ agonist group was injected intraperitoneally with rosiglitazone (10 mg/kg [24], MedChemExpress, USA) once daily for seven days. The hypoxia injury group was injected with an equal volume of 0. 9% saline once a day for seven days. The normal control group was under normoxic conditions in a standard environment equivalent to an altitude of about 600 m. At the end of the experiment, 1.5 mL of blood was collected *via* tail veins without anesthesia and the animals were euthanized with an overdose of sodium pentobarbital (150 mg/kg administered intraperitoneally). Lack of heartbeat and breathing for >5 min were considered to indicate animal death. A body weight loss of >15% with a decreased ability to consume food and water was used as the humane endpoint. Finally, the bilateral kidney was collected for histological examination.

### 2.3. Methods

#### 2.3.1. Renal function test

According to the manufacturer’s protocols, rat serum creatinine (SCr) and blood urea nitrogen (BUN) renal function indices were measured by sarcosine oxidase assay and urease assay, respectively, using relevant assay kits (Nanjing Jiancheng Institute of Biological Engineering, Nanjing, China).

#### 2.3.2. Renal histopathological examination

Paraffin-embedded kidney tissues were cut into 4-μm-thick sections. For hematoxylin and eosin (H&E) staining, sections were dehydrated with gradients of xylene and ethanol and stained with hematoxylin for five minutes at room temperature. After rinsing, sections were incubated with eosin for five minutes at room temperature. For periodic acid-Schiff (PAS) staining, sections were dehydrated with gradient dewaxing in xylene and ethanol and stained with PAS stain kit (Servicebio, Technology Co. Ltd., Wuhan, China). For Masson staining, sections were stained with Masson stain kit (Servicebio), rinsed with 1% glacial acetic acid for differentiation, and then dehydrated with anhydrous ethanol and sealed with neutral gum. Observations were made under a light microscope.

#### 2.3.3. Immunofluorescence

Paraffin sections were dewaxed in xylene, dehydrated in a graded ethanol solution, and then placed in ethylenediaminetetraacetic acid antigen repair buffer (pH 8.0) for antigen repair. Sections were cooled to room temperature and rinsed three times with phosphate buffered saline (PBS) (pH 7.4) for 5 min each. Circles were drawn around the tissue with a histochemical pen and the PBS was shaken off and closed with 3% BSA for 30 min. Primary antibody nuclear factor kappa-B p65 (NF-κB p65) (1:200; Servicebio) was added to the sections and incubated overnight at 4 °C in the refrigerator. Sections were washed with PBS, shaken dry slightly and Cy3 goat anti-rabbit secondary antibody (1:300; Servicebio) was added dropwise in the circle and incubated for 50 min at room temperature, protected from light. 4′,6-diamidino-2-phenylindole (DAPI) stain was used to stain the nuclei and samples were incubated for 10 min at room temperature protected from light. Sections were washed with PBS, an autofluorescence quencher was added and the sections were sealed with an anti-fluorescence quenching sealer (Servicebio). Images were acquired using a 3 D HISTECH digital section scanning system (Panoramic, 3 D HISTECH, Hungary).

#### 2.3.4. Tunel assay

A one-step TUNEL *in situ* apoptosis detection kit (Elabscience Biotechnology Co. Ltd., China) was used for the assay. Paraffin sections were dewaxed and hydrated, rinsed three times with PBS; proteinase K working solution was added dropwise and reacted at 37° for 20 min. The permeabilized samples were rinsed with PBS, and then 100 μL of TdT equilibration buffer was added to each sample and reacted at 37 °C for 10–30 min; 50 μL of labeling working solution was added dropwise, and the sections were placed in a wet box for 60 min at 37 °C and protected from light. The sections were rinsed with PBS, re-stained with DAPI working solution, rinsed again with PBS, and sealed. Three to five different visual fields were randomly selected to count the TUNEL positive cells and the total number of cells in each visual field. Apoptosis rate is expressed as apoptotic index (number of apoptotic cells/total number of cells).

#### 2.3.5. Quantitative real-time polymerase chain reaction (qRT-PCR)

Total ribonucleic acid (RNA) was extracted from renal tissue, and mRNA was reverse transcribed into cDNA (Nanjing Novozymes Biotechnology Co. Ltd., China). Primers were designed and synthesized by Sangon Biotech (Shanghai, China) (Table 1). The PCR reaction system consisted of 2 × ChamQ Universal qPCR SYBR Master Mix 10.0 Μ l; 0.4 μL each of forward and reverse primers (10 μmol/L), 1.0 μL cDNA, and 8.2 μL enzyme-free water. The reaction program was as follows: predenaturation at 95 °C for 30 s; 40 cycles at 95 °C, 10 s, 60 °C, 34 s; melting curve: 95 °C, 15 s, 60 °C, 1 min, 95 °C, 15 s. The relative expression of the target gene was calculated by 2^−△△CT^.

**Table 1.** List of primers used for quantitative real-time PCR analysis of kidney tissues.

Gene	Primer(5′-3′)	Length of product (bp)
PPARγ	For: CGCCAAGGTGCTCCAGAAGATG Rev: AGGGTGAAGGCTCATATCTGTCTCC	106
Bcl-2	For: TGGAGAGCGTCAACAGGGAGATG Rev: GTGCAGATGCCGGTTCAGGTAC	83
Bax	For: AGACACCTGAGCTGACCTTGGAG Rev: TTCATCGCCAATTCGCCTGAGAC	86
β-actin	For: TGTCACCAACTGGGACGATA Rev: GGGGTGTTGAAGGTCTCAAA	165

#### 2.3.6. Western blot

Rat kidney tissue was ground, and total protein was extracted with protein lysate. Protein concentration was detected by a bicinchoninic acid kit (Thermo Fisher Scientific, USA). After adding protein loading buffer and boiling for denaturation for 10 min, 20 μg of denatured protein was separated by 12% SDS-polyacrylamide gel electrophoresis and transferred to polyvinylidene fluoride membrane by wet transfer method. After defatted milk was blocked for 1.5 h at room temperature, it was incubated overnight at 4 °C in a primary antibody PPARγ (1:1000; Signalway Antibody, USA), Bax (1:1000; Proteintech, China), Bcl-2 (1:1000; Abcam, UK), NF-κB p65 and phospho-NF-κB p65 (1:1000; Cell Signaling Technology, USA) incubation box in a refrigerator shaker. Membranes were washed with tris buffered saline with tween thrice, for 10 min each time; horseradish peroxidase-labeled secondary antibodies (1:10000; Invitrogen31460) were added for one hour at room temperature in the dark. On the FluorChem M imaging system, the enhanced chemiluminescence reagent was dropped and exposed in the system. The results were analyzed using ImageJ software, and the ratio of the gray value of the target protein band to the gray value of the internal reference *β*-actin band was used as the relative expression of the target protein.

#### 2.3.7. Observation of mitochondrial structure

Renal cortical tissue of 1 mm³ size was cut and transferred to electron microscopy for fixation at 4° C. After rinsing with PBS (0.1 mol/L) three times for 15 min each time, they were then fixed with 1% osmic acid in the dark at room temperature for two hours and then rinsed with PBS three times again. Dehydration was performed with different concentrations of ethanol, followed by step-by-step dehydration with different concentrations of acetone, and osmotic embedding and warming polymerization with an 812-embedding medium. Ultrathin sections of 60–80 nm were made using an ultramicrotome and double stained with 2% uranyl acetate saturated alcohol solution and 2.6% lead citrate solution, and renal ultrastructure was observed using a transmission electron microscope (HT7800/HT7700; Hitachi).

### 2.4. Statistical analysis

SPSS 27.0 software was used for statistical analysis, and the data results were expressed as means ± standard deviation. All experiments were repeated at least three times. When the data obeyed a normal distribution, a one-way analysis of variance was used to compare multiple groups, followed by the LSD test (when the variances were homogeneous) or Dunnett’s T3 test (when the variances were heterogeneous), and *p* < .05 was considered statistically significant.

## 3. Results

### 3.1. Rosiglitazone improves hypoxia renal injury

**Figure 1.**
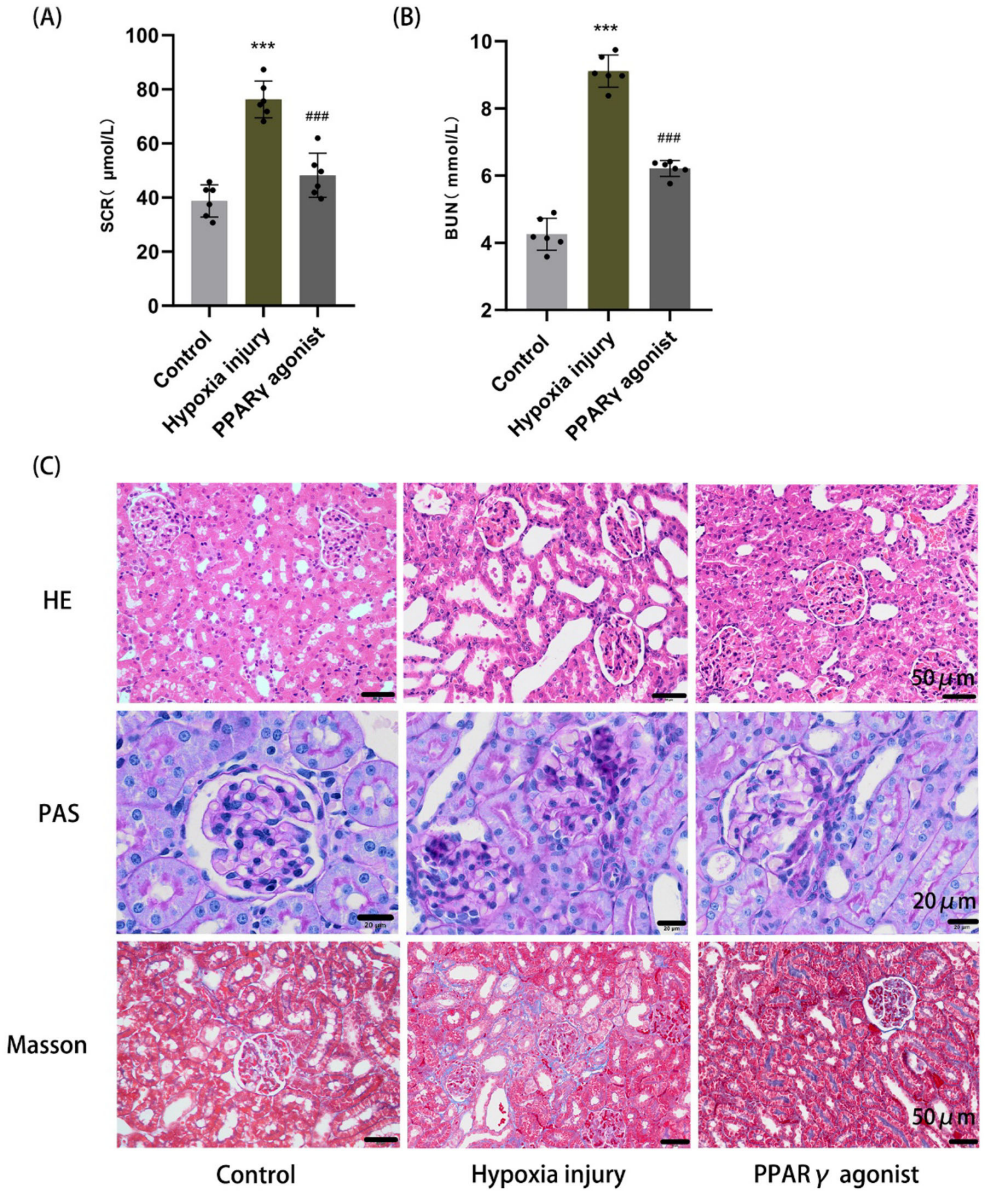
Renal function and histopathological changes in rats. **(A, B)** Detection of serum creatinine (SCr) and blood urea nitrogen (BUN) in rats by the sarcosine oxidase method and urease method, respectively. Data are expressed as mean ± SD, *n* = 6/group. ***p < .001 compared with the control group; *^###^p* < .001 compared with the hypoxia injury group. **(C)** Histopathological changes in the kidney, including glomeruli, tubules, and renal interstitial (HE, PAS, and Masson staining, magnification 400×).

Compared with the normal control group (SCr: 38.75 ± 5.96 μmol/L; BUN: 4.26 ± 0.48 μmol/L), the SCr and BUN levels were significantly higher in the hypoxia injury group (SCr: 76.26 ± 6.79 μmol/L; BUN: 9.11 ± 0.48 μmol/L) (*p* < .001); while the PPARγ agonist group (SCr: 48.19 ± 8.19 μmol/L; BUN: 6.21 ± 0.24 μmol/L) had significantly lower SCr and BUN levels compared with the hypoxia injury group (*p* < .001) (Figure 1(A and B)). H&E staining showed that in the hypoxia injury group, tubular epithelial cells were detached and cytoplasm was loose and lightly stained, a large number of renal tubules were dilated, and a small amount of inflammatory cells infiltration was observed in renal interstitial; a little tubular epithelial cell detachment and partial tubular dilatation were observed in the PPARγ agonist group, but they were milder than those in the hypoxia injury group; no injury was observed in the renal tissue of the normal control group. PAS staining showed that cell debris in the tubular lumen, mesangial cells and matrix proliferated and some glomerular basement membranes were uneven in thickness in the hypoxia injury group, and the degree of the PPARγ agonist group was lighter than that of the hypoxia injury group. Masson staining showed a small amount of collagen fiber deposition in the renal interstitia of the hypoxia injury group, and no significant collagen fiber deposition was found in the normal control and PPARγ agonist groups (Figure 1(C)).

### 3.2. Rosiglitazone attenuates apoptosis in a PPARγ-dependent manner

**Figure 2.**
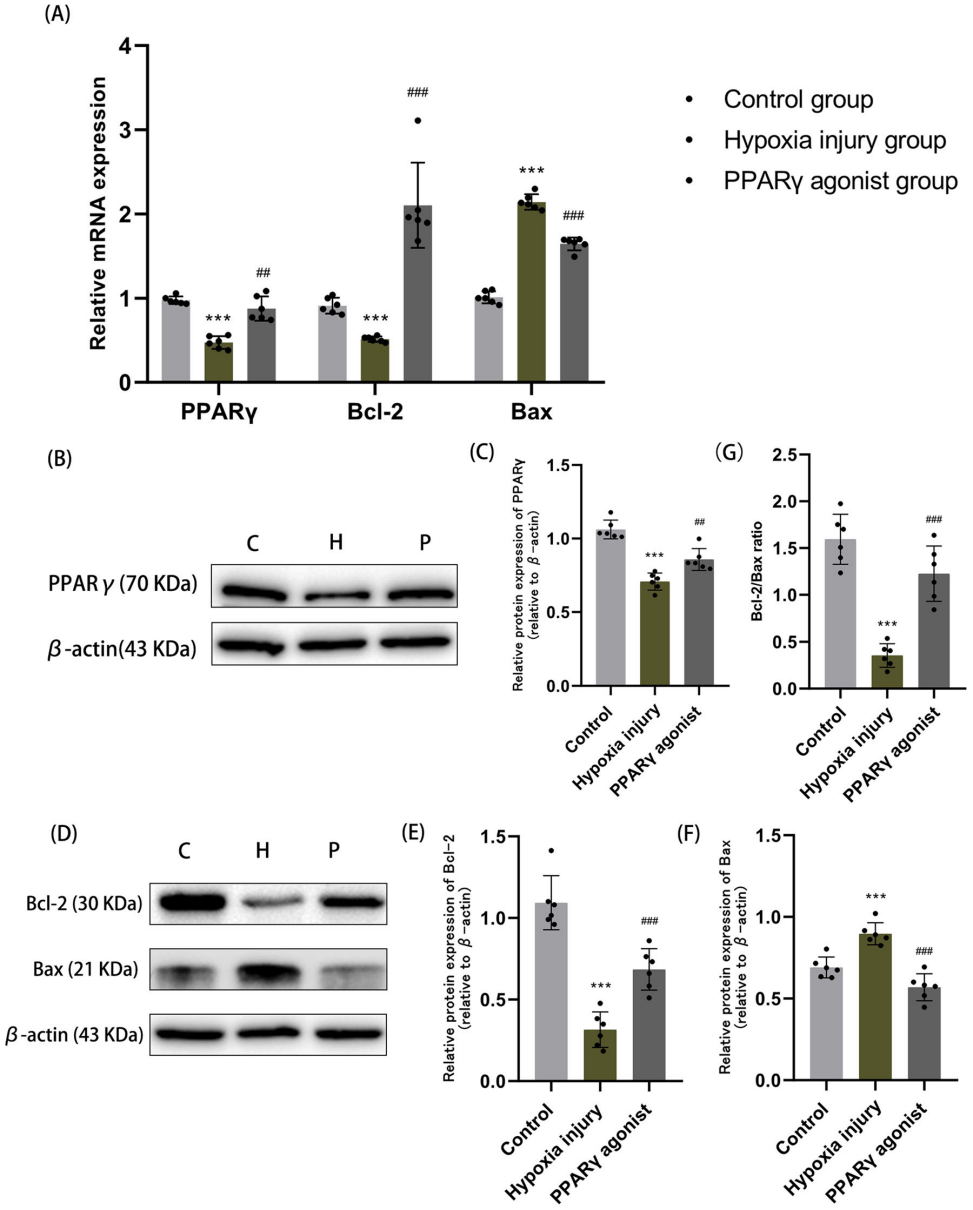
Expression of PPARγ and apoptosis-related factors Bcl-2 and Bax in renal tissues. **(A)** qRT-PCR analysis of mRNA expression of PPARγ, Bcl-2 and Bax in each group of kidney tissues. **(B, C)** Western blot analysis of PPARγ protein expression in renal tissue. **(D-F)** Western blot analysis of protein expression of apoptosis-related factors Bcl-2 and Bax. **(G)** The ratio of Bcl-2 to Bax (Bcl-2/Bax) in renal tissue, as determined by Western blot analysis. Data are expressed as mean ± SD, *n* = 6/group. ***p < .001 compared with the control group; *^##^p* < .01, *^###^p* < .001 compared with the hypoxia injury group. C: Control group; H: Hypoxia injury group; P: PPARγ agonist group.

Compared with normal controls, the protein and mRNA expression of PPARγ significantly decreased after hypoxia; however, following administration of a PPARγ agonist, these levels significantly raised (all *p* < .01) (Figure 2(A–C)). Additionally, we observed that Bcl-2 expression significantly decreased, Bax expression levels significantly increased, and the Bcl-2/Bax ratio significantly decreased in the hypoxia injury group compared with the normal control group; the differences were statistically significant (all *p* < .01). Following rosiglitazone administration, Bcl-2 expression significantly increased, Bax expression levels significantly decreased, and the Bcl-2/Bax ratio significantly increased, and the differences were statistically significant (all *p* < .01) (Figure 2(D–G)). This suggests that the increased expression of PPARγ can decrease the expression of pro-apoptotic factor Bax, increase the expression of anti-apoptotic factor Bcl-2, reduce apoptosis, and thus improve hypoxia renal injury.

### 3.3. Rosiglitazone improves hypoxia renal injury by regulating NF-κB signaling pathway

**Figure 3.**
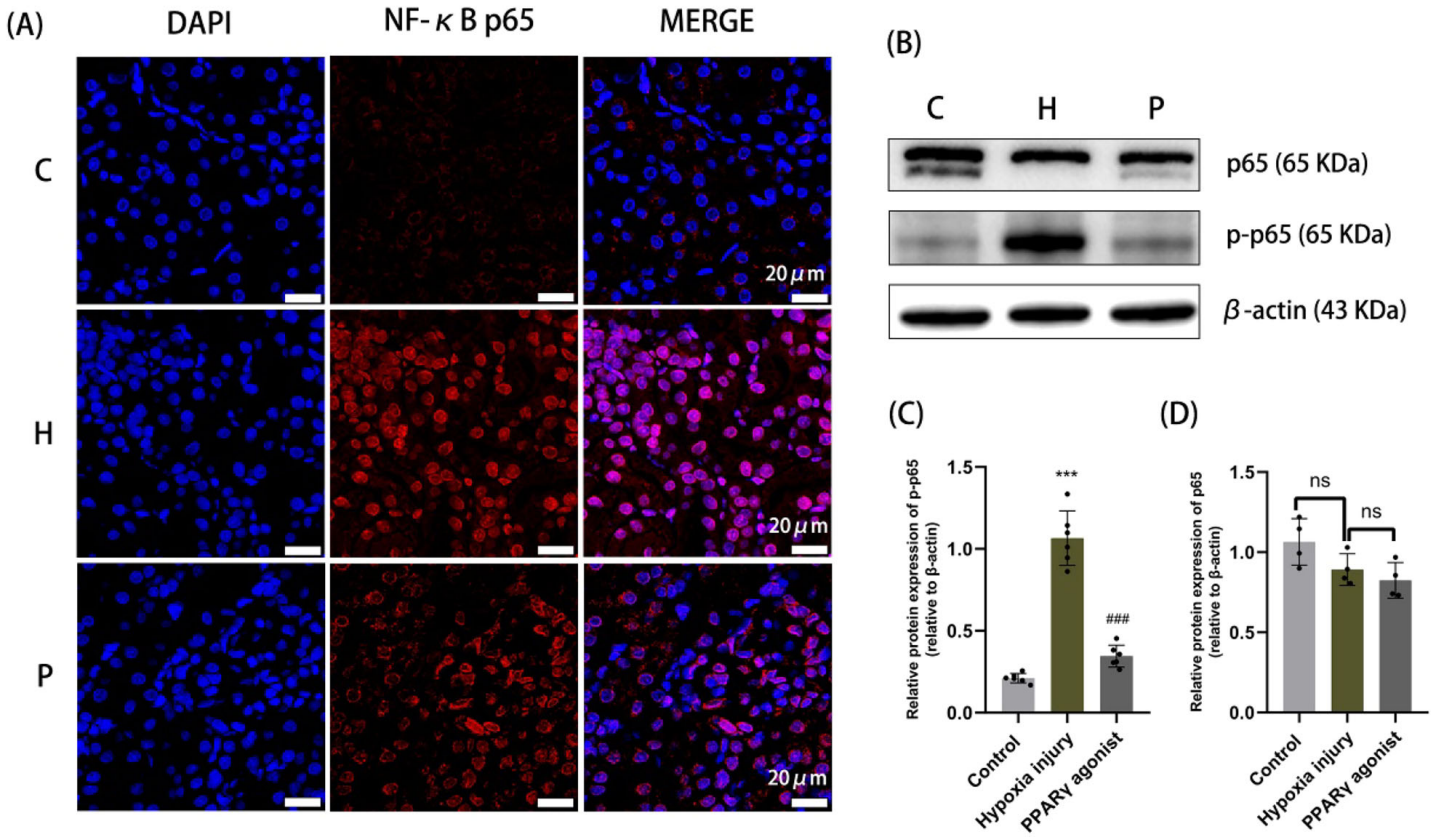
Localization and protein expression of NF-κB in renal tissue. **(A)** Immunofluorescence staining with paraffin sections, with NF-κB p65 shown in red and DAPI nuclear staining shown in blue (magnification 630×); **(B-D)** Protein expression of NF-κB p65 in rat kidney detected by Western blot analysis. Data are expressed as mean ± SD, *n* = 6/group. ***p < .001 compared with the control group; *^###^p* < .001 compared with the hypoxia injury group. C: Control group; H: Hypoxia injury group; P: PPARγ agonist group.

Immunofluorescence results showed that NF-κB in the normal control group was localized in the cytoplasm; whereas in the hypoxia injury group NF-κB was mostly localized in the nucleus, which suggested that the NF-κB signaling pathway was activated when NF-κB was transferred from the cytoplasm into the nucleus. However, NF-κB nuclear translocation decreased after administration of rosiglitazone (Figure 3(A)). In addition, western blot results showed that NF-κB p65 protein expression significantly increased after hypoxia and it decreased after rosiglitazone administration, which suggested that the up-regulation of PPARγ, inhibited the activation of the NF-κB pathway (Figure 3B-D).

### 3.4. Tunel analysis and mitochondrial morphological changes in renal tissue

**Figure 4.**
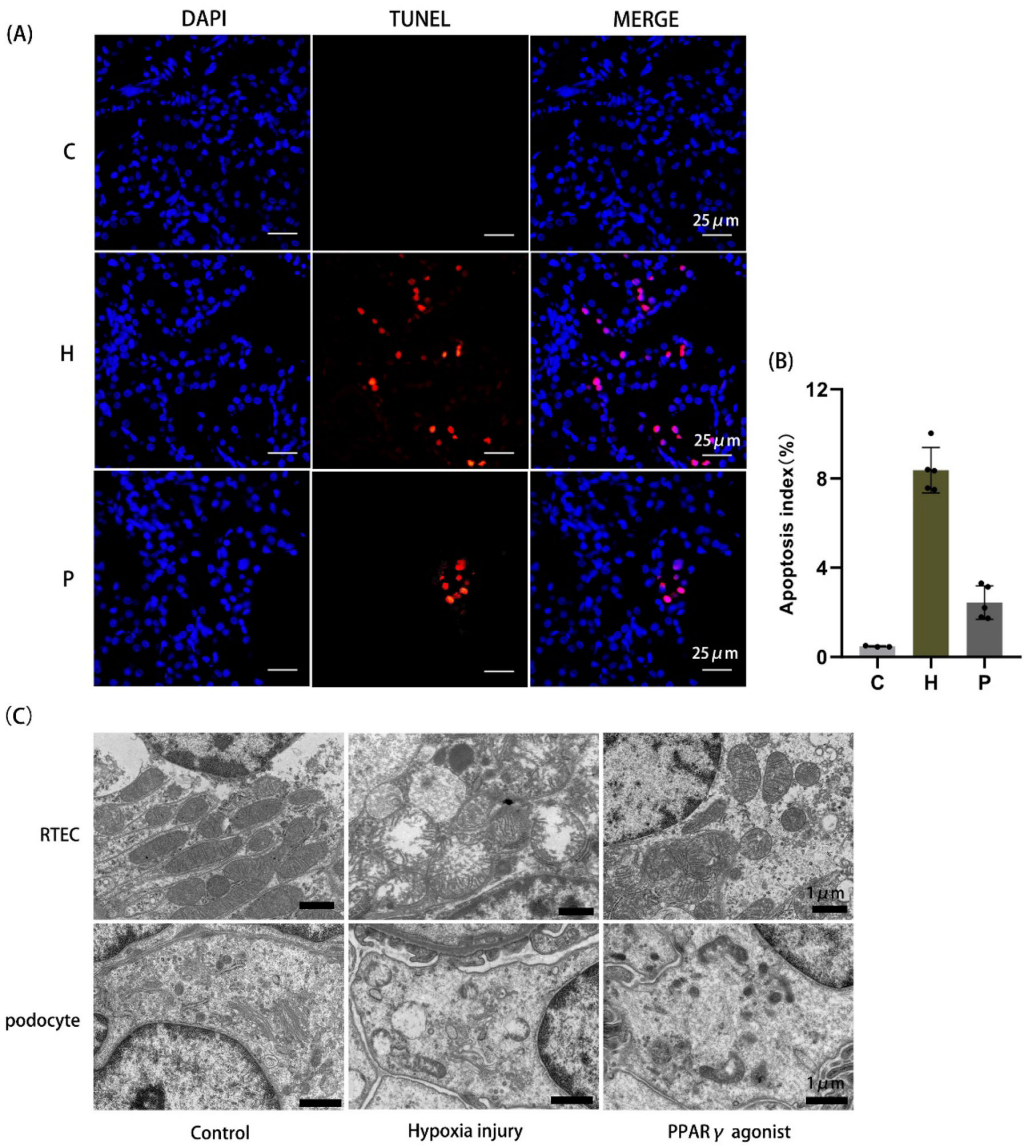
Detection of TUNEL apoptosis in renal tissue and observation of mitochondrial morphology by transmission electron microscopy. **(A)** TUNEL apoptosis detection, as shown by red light (400×). **(B)** Area occupied by TUNEL-positive cells in renal tissue, as determined by image analysis. **(C)** Changes in mitochondrial structure in renal tissue in each group observed by transmission electron microscopy (RTEC magnification 5000×; podocyte magnification 6000×). C: Control group; H: Hypoxia injury group; P: PPARγ agonist group.

Apoptotic cells increased after hypoxia compared to normal controls, whereas rosiglitazone reduced apoptosis (Figure 4(A and B)). Mitochondrial morphology of RTEC and podocytes was observed by transmission electron microscopy. In the normal control group, the number of mitochondria was abundant, the size was uneven, the cristae were intact, and the adventitia were smooth without swelling; in the hypoxia injury group, the number of mitochondria was reduced, the structure was destroyed, the mitochondria were severely swollen, and the cristae were broken. The degree of injury in the PPARγ agonist group was milder than that in the hypoxia injury group. The mitochondrial structure was normal in podocytes of the normal control group; mitochondria were severely swollen, the matrix was dissolved, and many cristae were broken and absent in the hypoxia injury group; mitochondrial damage was milder in the PPARγ agonist group than in the hypoxia injury group (Figure 4(C)).

## 4. Discussion

The high sensitivity of the kidney to hypoxia has led to increasing attention to hypoxia renal injury. Earlier models of hypoxia renal injury are mostly renal ischemia-reperfusion models. We found that this model could not simulate prolonged renal hypoxia, and the simulation of chronic hypoxia renal injury was extremely limited. Therefore, we proposed a model of hypobaric hypoxia to investigate the effects of hypoxia on the kidney of rats. In this study, the expression of PPARγ decreased, renal function was affected and renal tissue was damaged in rats after seven days of hypoxia exposure; renal injury induced by hypoxia was improved by the use of rosiglitazone, a PPARγ agonist.

Expression of PPARγ is observed in the renal medulla and interstitia, including podocytes, mesangial cells, and renal microvascular endothelial cells [25]. Given that a variety of kidney cells have endogenous PPARγ expression and activity, we hypothesize that PPARγ activation in the kidney may be critical for regulating renal function. The PPARγ agonist rosiglitazone attenuates acute kidney injury in septic rats [26], and another PPARγ agonist pioglitazone also attenuates cirrhosis-related renal dysfunction and chronic renal insufficiency caused by endotoxemia [27]. In recent years, PPARγ was found to be associated with hypoxia injury [28,29]. During hypoxia, oxidative stress pathways can activate various transcription factors such as PPARγ, NF-κB, and HIF-1α [30]. Blanquicett et al. found that PPARγ activity and expression in vascular endothelial cells were suppressed under hypoxia conditions by inhibiting redox-regulated transcription factors [31]. Our previous study found that PPARγ and mitophagy are involved in hypoxia/reoxygenation-induced renal tubular epithelial cells injury [29]. Consistent with these results, in this study, we showed that after hypoxia, the expression of PPARγ in rats significantly decreased, SCr and BUN significantly increased, and renal histopathology showed significant damage; by contrast, after administration of rosiglitazone, a PPARγ agonist, the expression of PPARγ significantly increased, and renal injury improved, which indicated that PPARγ may be involved in hypoxia renal injury. However, in this study, the PPARγ agonist rosiglitazone was protective in the kidney, but its exact site of action is unknown.

In addition, PPARγ is closely related to apoptosis in the mitochondrial pathway. the PPARγ agonist GL516 reduces oxidative stress and apoptosis in rat astrocyte cell lines [32]. PPARγ activation prevents cardiac insufficiency by inhibiting apoptosis and necrosis [33], and by regulating PPARγ expression, it induces Bcl-2, Bax, and cytochrome-c expression changes and attenuates cardiomyocyte apoptosis [34]. Recent studies have found that PPARγ may exert renal protective effects by inhibiting apoptosis. In this route, Bax and Bcl-2 act as the main molecules of mitochondria-associated proteins, in which maintaining the ratio of Bcl-2 to Bax prevents apoptosis; however, an imbalance in this ratio, which enhances mitochondrial permeability, leads to DNA fragmentation and cell death [35,36]. Wen LL et al. reported that the increase in PPARγ expression prevented apoptosis and decreased the levels of Bcl-xS and caspase-3 in rat renal tubular epithelial cells [21]. The results showed that after hypoxia, Bcl-2 expression decreased and Bax expression increased, and mitochondrial structure was severely damaged. The opposite was observed when rosiglitazone was administered, which suggests that rosiglitazone attenuates hypoxia-induced apoptosis in renal tissue. Moreover, inflammatory responses and oxidative stress induced by the mitochondrial pathway, such as changes in mitochondrial membrane potential and reactive oxygen species, can also contribute to kidney injury [37,38].

In this study, the expression of NF-κB was elevated in kidney tissue following hypoxia in rats. Therefore, we hypothesize that hypoxia-induced apoptosis in renal tissue may be mediated through the NF-κB signaling pathway. NF-κB is a group of structurally related protein families (Rel protein family), including RelA (p65), RelB, RelC, NF-κB1 (p50), and NF-κB2 (p52), of which p50 and p65 are the major components of NF-κB activity, and p65 is predominant among activated NF-κB subunits. NF-κB is widely involved in cellular regulation, immune response, inflammatory response, and other biological processes [39]; it is also an important signaling molecule downstream of PPARγ and is regulated by the inactivation of PPARγ [40,41]. Upregulation of PPARγ expression in a variety of pathological states can lead to a decrease in NF-κB activation levels [42]. Ju et al. reported that NF-κB nuclear levels were significantly lower after treatment with optimized PPARγ agonists [43]. Bergenin attenuates H_2_O_2_-induced oxidative stress and apoptosis in human nucleus pulposus cells by activating PPARγ and inhibiting the NF-κB pathway [44]. In addition, through NF-κB interaction with multiple molecules in the mediated apoptotic signaling pathway through the target gene products Bcl-2 and Bcl-xL, which inhibit the endogenous apoptotic pathway [45], and through the upregulation of the apoptotic protein cytosolic inhibitor-1/-2 and FLICE-like inhibitory protein expression to suppress the exogenous apoptotic pathway [46], thus affecting the disease response process.

There are several limitations to this study. First, our study was short of mechanistic studies about PPARγ inhibitors in hypoxia injury, and we plan to carry out subsequent experiments to investigate it. In addition, the mechanism by which rosiglitazone attenuates hypoxia kidney injury may also be related to inflammation and oxidative stress. We will conduct more in-depth research in this area to shed light on this issue.

## 5. Conclusion

In conclusion, the renal condition of hypoxia rats was improved after administration of rosiglitazone, a PPARγ agonist, which may be achieved by rosiglitazone through inhibition of NF-κB signaling pathway in a PPARγ-dependent manner, attenuating mitochondrial pathway-mediated renal apoptosis. This has important implications for PPARγ as a new drug for the treatment of hypoxia kidney injury.

## Data Availability

The data of this study can be obtained from the corresponding author upon reasonable request.
